# Double Whammy: Subacute Stent Thrombosis While Being Adherent to Dual Antiplatelet Therapy Including Ticagrelor, Followed by Multiple Coronary Artery Aneurysms in a COVID-19 Patient

**DOI:** 10.7759/cureus.21908

**Published:** 2022-02-04

**Authors:** Navdeep S Sidhu, Sumandeep Kaur

**Affiliations:** 1 Department of Cardiology, Guru Gobind Singh Medical College & Baba Farid University of Health Sciences, Faridkot, IND; 2 Faculty of Nursing Sciences, Baba Farid University of Health Sciences, Faridkot, IND

**Keywords:** sars-cov-2 (severe acute respiratory syndrome coronavirus -2), coronavirus disease 2019, percutaneous coronary intervention, dual antiplatelet therapy, ticagrelor, coronary artery aneurysm, stent thrombosis, covid-19, acute coronary syndrome

## Abstract

Coronavirus disease 2019 (COVID-19), although predominantly a respiratory illness, can have important cardiovascular implications, which include the development of myocardial injury/myocarditis, acute coronary syndromes, arrhythmias, pericarditis, and the occurrence of arterial and venous thrombosis. We describe a rare case of a middle-aged COVID-19 patient who developed sub-acute stent thrombosis after implantation of second-generation drug-eluting stents (DES) despite being adherent to dual antiplatelet therapy including ticagrelor and who subsequently developed multiple coronary artery aneurysms within a few weeks of the DES implantation.

## Introduction

The development and clinical use of drug-eluting stents (DES) in 2000s was a major breakthrough in the field of interventional cardiology. First-generation DES remarkably reduced the rates of in-stent restenosis and consequent target-lesion revascularization, as compared to the bare-metal stents. However, a major safety concern regarding stent thrombosis emerged with the use of first-generation DES, with its attendant high rates of myocardial infarction (MI) and mortality. To overcome these shortcomings of first-generation DES, second-generation DES have been developed with focus on the use of thinner stent struts and biocompatible or biodegradable polymers. Although second-generation DES have been associated with improved outcomes as compared to the first-generation DES, stent thrombosis still remains a significant problem in the contemporary clinical practice even with the use of potent anti-platelet agents like ticagrelor and prasugrel [1].

The occurrence of coronary artery aneurysms (CAA) after percutaneous coronary intervention (PCI) is a rare event, with various possible etiological factors including mechanical trauma during PCI, coronary dissection, balloon/stent oversizing, high-pressure balloon dilatation, use of atherectomy/laser angioplasty, stent mal-apposition, delayed re-endothelization, and local hypersensitivity reaction to the DES polymer [2]. Non-PCI-related causes of CAA include atherosclerosis, vasculitis syndromes (like Kawasaki’s disease, Takayasu's arteritis, polyarteritis nodosa, systemic lupus erythematosus), connective tissue disorders (like Marfan’s syndrome, Ehlers-Danlos syndrome), and infections (like bacterial, fungal, HIV) [3].

We present an interesting case of a middle-aged male who developed subacute stent thrombosis while being on dual antiplatelet therapy (DAPT) including ticagrelor, during an episode of coronavirus disease 2019 (COVID-19) and who subsequently developed multiple CAA at the site of previous stents.

## Case presentation

**Figure 1:**
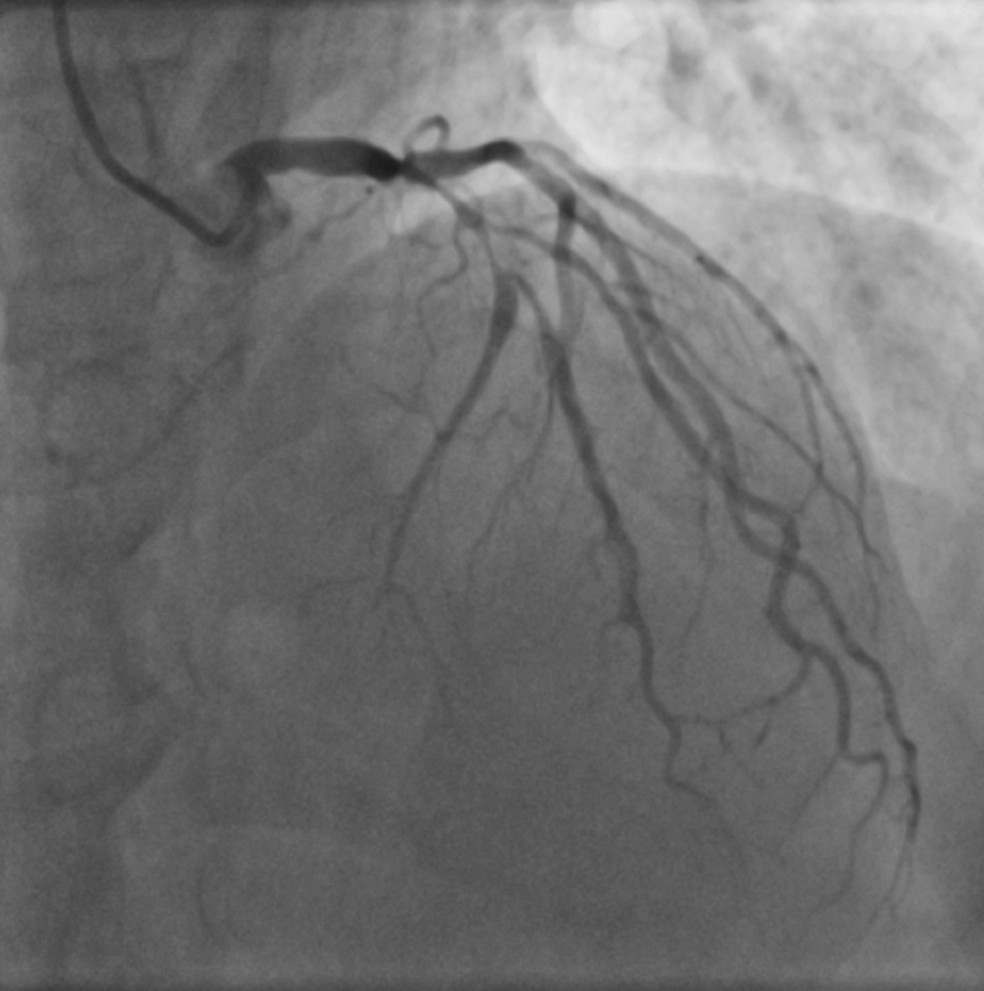
Coronary angiogram in anterior-posterior (AP) cranial view showing critical stenosis of left anterior descending artery (LAD)/diagonal bifurcation

**Figure 2:**
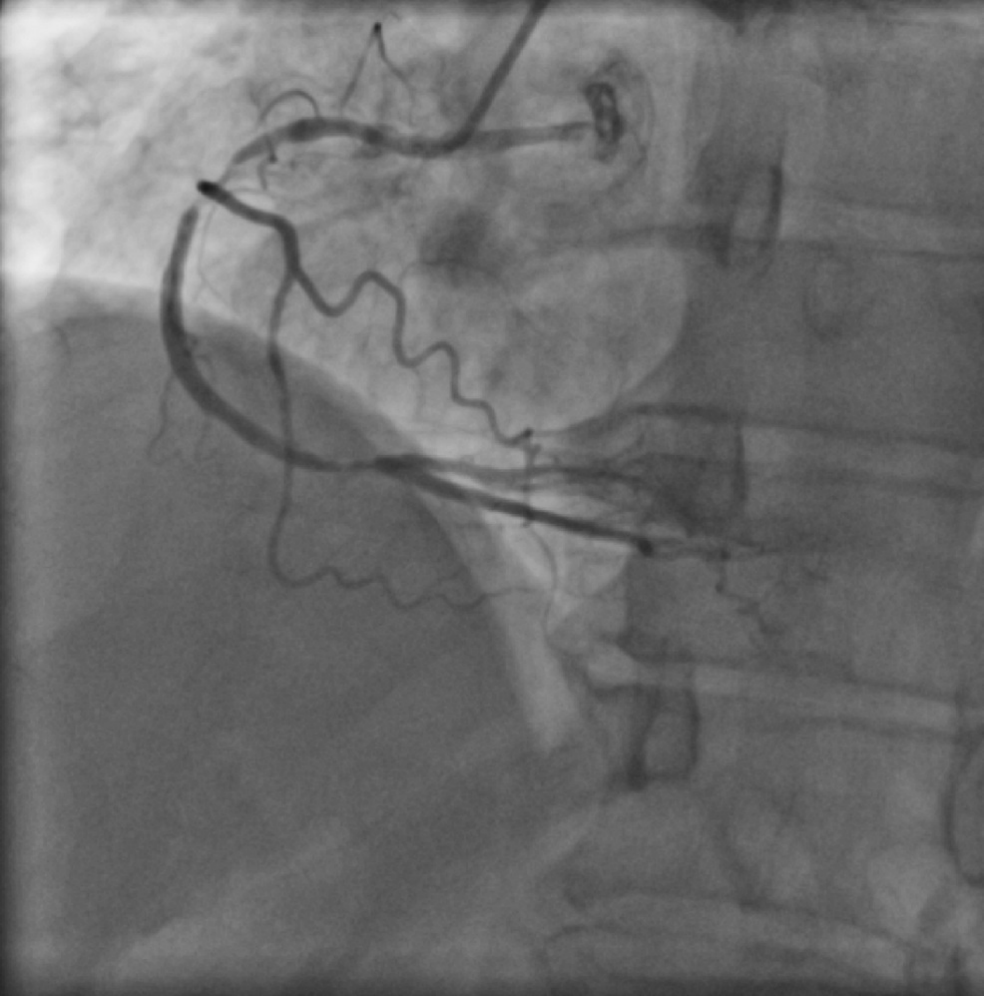
Coronary angiogram in left anterior oblique (LAO) view showing critical stenosis in mid and distal right coronary artery (RCA)

A 56-year-old non-hypertensive, non-diabetic, non-smoker, dyslipidemic male presented to the emergency department with acute chest pain of 5 hours duration. His electrocardiography (ECG) revealed non-specific ST-T changes and he had an elevated troponin levels of 0.12 ng/ml (N < 0.01 ng/ml). There was no regional wall motion abnormality on echocardiography and he had a normal left ventricular ejection fraction (LVEF). A diagnosis of non-ST elevation acute coronary syndrome was established and he was scheduled for coronary angiography (CAG) after loading doses of aspirin, ticagrelor, and atorvastatin. On CAG he was found to be having critical stenosis of left anterior descending (LAD)/diagonal bifurcation (Figure 1, Video 1), and right coronary artery (RCA) (Figure 2, Video 2).

**Video 1:** Coronary angiogram in anterior-posterior (AP) cranial view showing critical stenosis of left anterior descending artery (LAD)/diagonal bifurcation

**Video 2:** Coronary angiogram in anterior-posterior (AP) cranial view showing critical stenosis in mid and distal right coronary artery (RCA)

**Figure 3:**
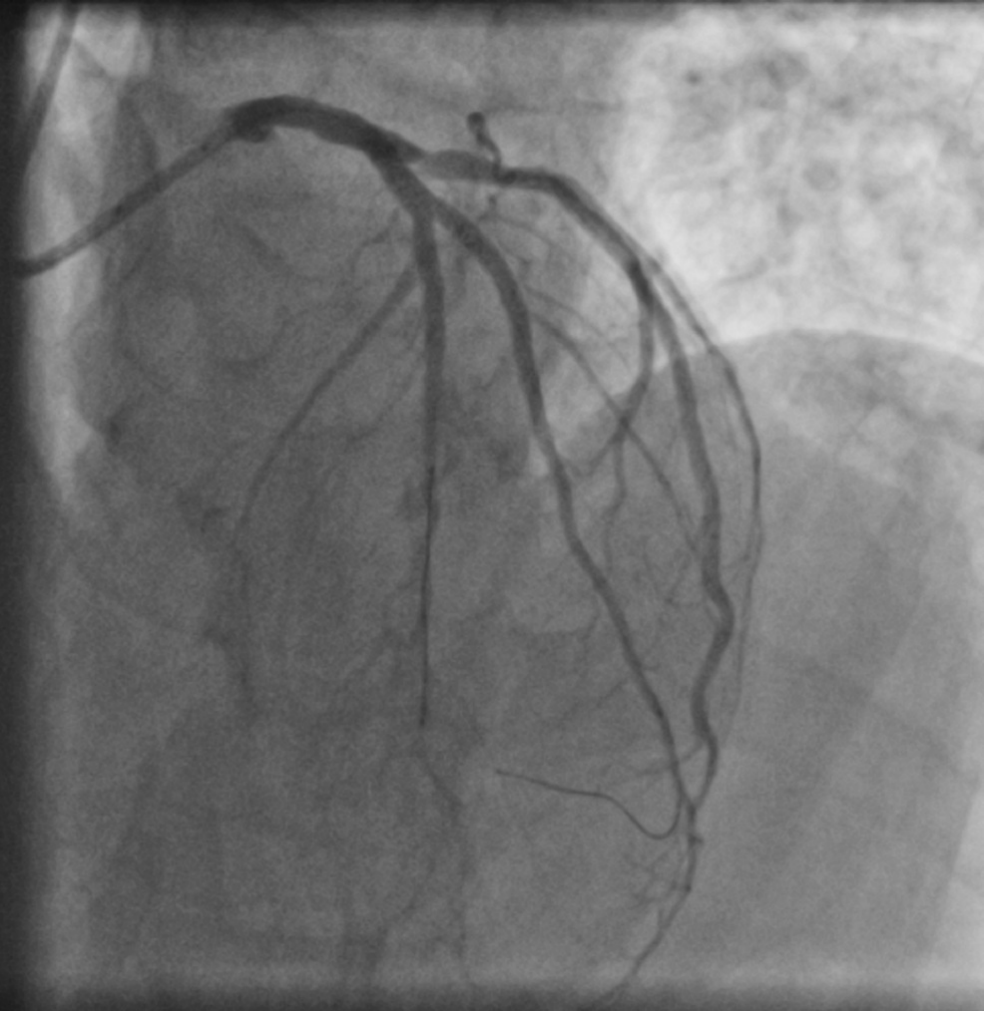
Coronary angiogram in left anterior oblique (LAO) cranial view after left anterior descending artery (LAD)/diagonal bifurcation stenting showing well-deployed stents

Immediate PCI was planned in view of critical stenosis and significantly elevated troponin levels. LAD/diagonal stenosis was treated with implantation of two everolimus-eluting stents (2.75 × 28 mm Xience Prime, 2.75 × 38 mm Xience Prime) at nominal pressures using double kissing crush technique. Post dilatation was done using 2.75 × 12 mm and 2.75 × 15 mm non-compliant balloons inflated at high pressures, followed by final kissing balloons' inflations with same balloons and proximal optimization using 3.5 × 12 mm non-compliant balloon (Figure 3, Video 3).

**Video 3:** Coronary angiogram in anterior-posterior (AP) cranial view after left anterior descending artery (LAD)/diagonal bifurcation stenting showing well-deployed stents with thrombolysis in myocardial infarction (TIMI) III flow

**Figure 4:**
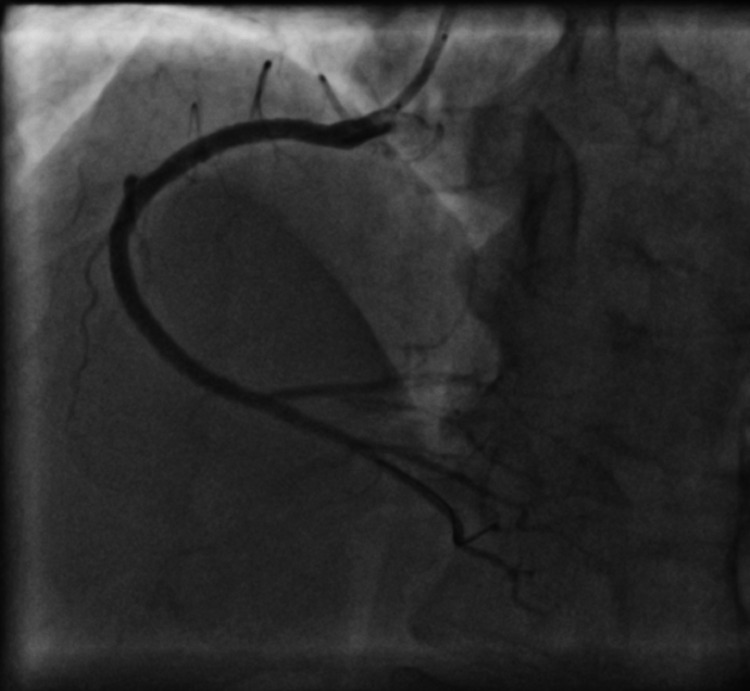
Coronary angiogram in left anterior oblique (LAO) view after right coronary artery (RCA) stenting showing well-deployed stents

RCA was treated using two overlapping everolimus-eluting stents (2.75 × 38 mm Xience Prime and 3 × 48 mm Xience Xpedition) followed by high-pressure post-dilatation with 3 × 15 mm and 3.25 × 15 mm non-compliant balloons (Figure 4, Video 4).

**Video 4:** Coronary angiogram in left anterior oblique (LAO) view after right coronary artery (RCA) stenting showing well-deployed stents with thrombolysis in myocardial infarction (TIMI) III flow

**Figure 5:**
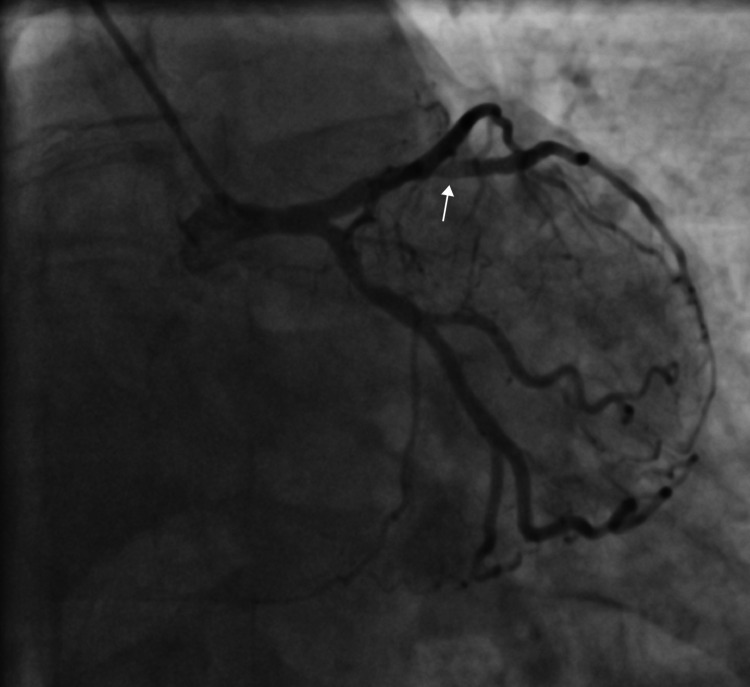
An anterior-posterior (AP) caudal view angiogram showing large thrombus at the origin of major diagonal (arrow)

**Figure 6:**
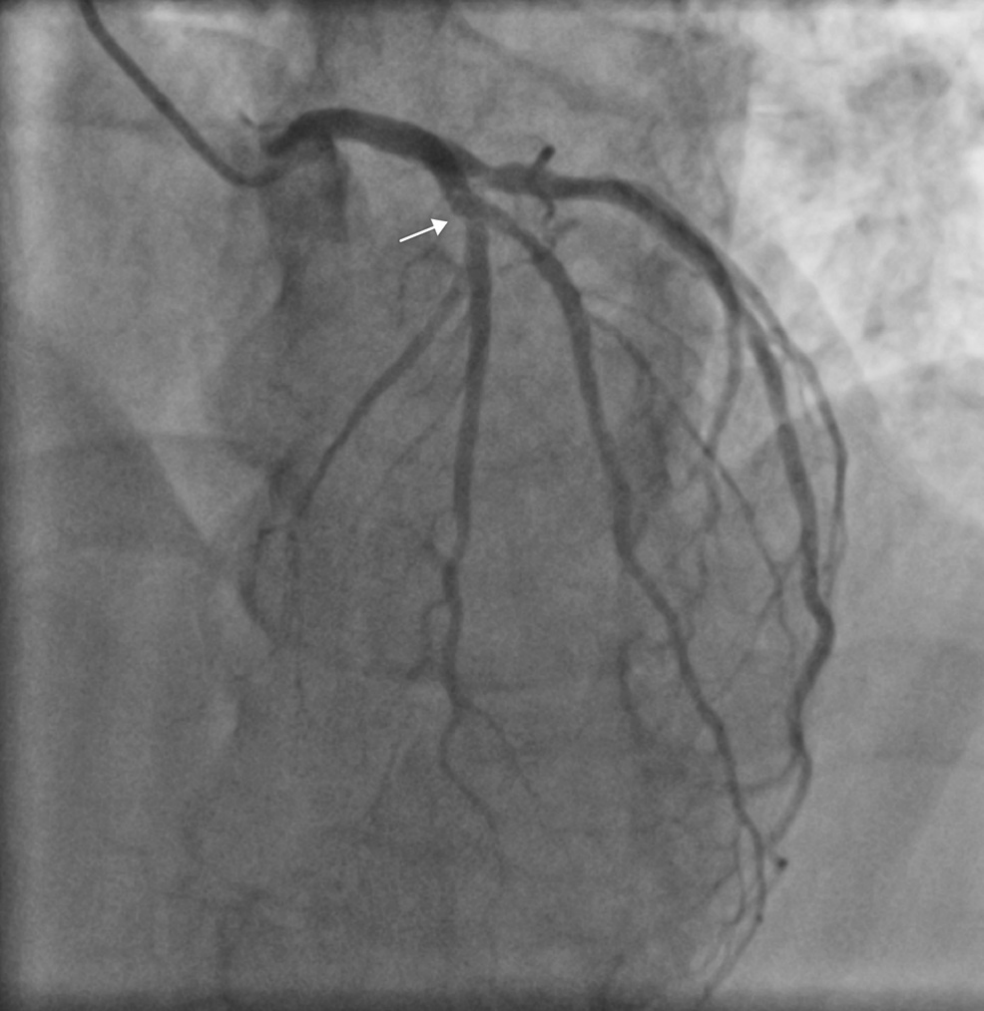
A left anterior oblique (LAO) cranial view angiogram showing visible thrombi in both left anterior descending artery (LAD) and diagonal branch near the bifurcation zone (arrow)

At the end of the procedure, all stents were well expanded with thrombolysis in myocardial infarction (TIMI) III flow and there was no angiographic evidence of any residual stenosis or edge dissections. As this case was performed at the peak of a COVID-19 wave in the region, a COVID-19 reverse transcriptase polymerase chain reaction (RT-PCR) was performed prior to the procedure, as mandated by the institutional protocol and it was found to be negative. Post-procedure stay of the patient was unremarkable and he was discharged after two days in an asymptomatic condition on DAPT (aspirin 75 mg OD and ticagrelor 90 mg BID) besides other medicines. Eight days after discharge, the patient again presented to the emergency department with acute chest pain. His ECG showed lateral wall ST elevation MI with ST elevation in leads I and aVL. Patient had good compliance with the medications and denied skipping any of the doses. In view of odd-hour presentation and non-availability of 24×7 catheterization laboratory services due to COVID-19 pandemic, he was thrombolyzed with reteplase. Simultaneously, ticagrelor was switched to prasugrel including a loading dose of 60 mg. He became asymptomatic within an hour of thrombolysis. Echocardiography revealed hypokinesia of anterolateral and lateral segments with an LVEF of 45%. During this admission, his COVID-19 RT-PCR was found to be positive, although he was afebrile, had no respiratory symptoms, and had a normal SpO_2_. He reported the occurrence of generalized body weakness and fatigue since last three to four days. CAG was performed on the next day and it showed features of stent thrombosis with a 20-30% thrombotic stenosis in LAD near the origin of the diagonal branch and a 70-75% thrombotic stenosis in ostio-proximal diagonal (Figure 5, Figure 6, Video 5, Video 6).

**Video 5:** An anterior-posterior (AP) caudal view angiogram showing large thrombus at the origin of major diagonal

**Video 6:** A left anterior oblique (LAO) cranial view angiogram showing visible thrombi in both left anterior descending artery (LAD) and diagonal branch near the bifurcation zone

**Figure 7:**
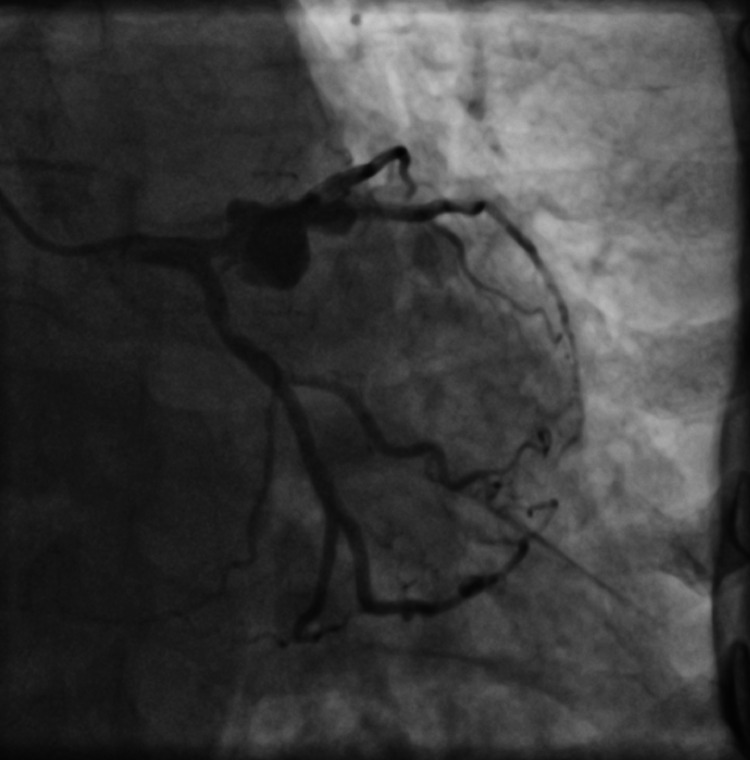
An anterior-posterior (AP) caudal view angiogram showing multiple aneurysms in the left anterior descending artery (LAD) and the diagonal branch at the sites of bifurcation stenting

**Figure 8:**
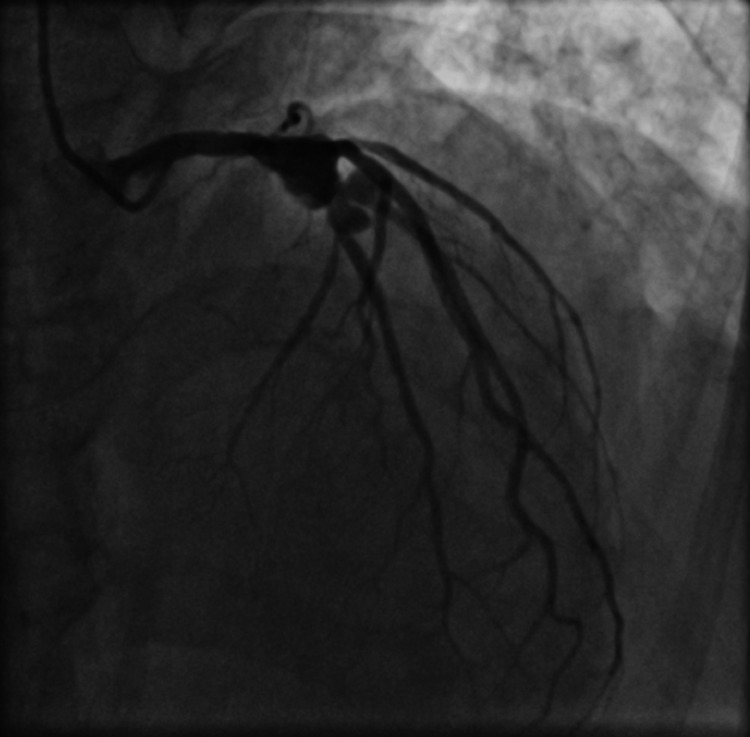
An anterior-posterior (AP) cranial view angiogram showing multiple aneurysms in the left anterior descending artery (LAD) and the diagonal branch at the sites of bifurcation stenting

RCA stents were patent with no evidence of thrombosis or re-stenosis. As there was TIMI III flow in LAD and diagonal and the patient was asymptomatic presently and had already undergone complex stenting procedure in the past, we decided for medical management in the form of DAPT (aspirin and prasugrel) along with enoxaparin (1 mg/kg s/c 12 hourly). Intra-coronary imaging could not be performed due to logistic limitations imposed by the COVID-19 pandemic. Blood investigations performed during this hospital admission revealed a normal hemogram, normal renal and liver function tests, and a normal chest X-ray. His peak troponin level during the admission was 1.8 ng/ml (N < 0.01 ng/ml). An increase in inflammatory markers was noted during the admission with peak levels of high-sensitivity C-reactive protein of 10.8 mg/l (N < 1 mg/l), serum ferritin of 828 μg/l (N 30-400 μg/l), and D-dimer of 1387 ng/ml (N < 500 ng/ml). Platelet function tests could not be performed due to non-availability at that time. In view of minimal symptoms related to COVID-19, patient was not administered any specific medications for this illness and a close clinical observation was advised. The patient remained asymptomatic during his subsequent stay in the hospital and was discharged on DAPT (aspirin and prasugrel) and dabigatran 110 mg BID after five days of enoxaparin, with a plan to perform a check-angiogram after a few weeks. An elective angiogram was performed after nearly six weeks of the DES implantation, and to our surprise, a large saccular aneurysm was seen in the stented segment of proximal LAD prior to the bifurcation and two smaller aneurysms were noted in mid LAD and major diagonal in the stented segments just after the bifurcation (Figure 7, Figure 8, Video 7, Video 8).

**Video 7:** An anterior-posterior (AP) caudal view angiogram showing multiple aneurysms in the left anterior descending artery (LAD) and the diagonal branch at the sites of bifurcation stenting

**Video 8:** An anterior-posterior (AP) cranial view angiogram showing multiple aneurysms in the left anterior descending artery (LAD) and the diagonal branch at the sites of bifurcation stenting

There was no angiographic evidence of intracoronary thrombus or flow-limiting lesions. RCA angiography was unremarkable. The patient was counselled regarding the possible need of surgical or percutaneous treatment for aneurysms, but he was not willing for any further intervention and wanted to continue with medical management only. Qualitative tests for anti-nuclear antibody and rheumatoid factor were found to be negative. His oral anticoagulation therapy was stopped and he was discharged on DAPT (aspirin and prasugrel), besides angiotensin-converting enzyme inhibitors, statins, and beta blockers. The patient is currently on medical therapy and is asymptomatic at one-year telephonic follow-up. However, we have not been able to convince him to undergo repeat angiography or imaging studies to re-assess the status of his coronary aneurysms.

## Discussion

We describe a rare case of a patient who developed subacute stent thrombosis while being on DAPT including ticagrelor therapy during an episode of COVID-19 illness and who subsequently developed multiple CAA within a few weeks. We believe that both these complications of PCI are probably related to the COVID-19 illness, as this illness has been shown to be associated with hypercoagulability and an hyperinflammatory state in the body, which could be responsible for the development of stent thrombosis and, subsequently, CAA.

Although COVID-19 usually presents with respiratory symptoms and signs, various cardiovascular manifestations including myocardial injury/myocarditis, arrhythmias, MI, and thromboembolism have been reported since the outbreak of this pandemic [4]. The occurrence of various types of stent thrombosis, ranging from acute to very late stent thrombosis, has been reported in patients with COVID-19 illness [5-11]. Pathophysiologically, a heightened systemic inflammatory response and a hypercoagulable state associated with the COVID-19 illness have been implicated as the major causative factors for increased incidence of both arterial and venous thrombosis during this illness. Also, severe acute respiratory syndrome coronavirus 2 (SARS-CoV-2) has been implicated in the causation of endothelitis, endothelial injury, endothelial dysfunction, and microcirculatory impairment [12]. Additionally, the presence of a coronary stent acts as a local stasis factor, thus completing the Virchow’s triad. In our case, we believe that the patient’s COVID-19 illness and a hyperinflammatory and hypercoagulable state associated with it played a major role in the development of stent thrombosis despite being compliant to DAPT including ticagrelor. A significant limitation of our case is that an intravascular imaging study could not be performed due to logistic limitations in wake of COVID-19 pandemic, and also, platelet reactivity testing was not available at that time. Although mechanical factors related to the PCI procedure, like stent under expansion, could be responsible for stent thrombosis, we believe that this cause was highly unlikely in our case as the post-procedure angiograms in two orthogonal views demonstrated well-expanded stents with no evidence of edge dissections or residual stenosis. Also, the asymptomatic status of the patient after his COVID-19 illness makes this possibility highly unlikely. The occurrence of stent thrombosis due to ticagrelor resistance has been reported rarely in the literature [13-16], and could have been contributory toward the development of stent thrombosis in our case, but the occurrence of true resistance to ticagrelor therapy is still debated [17]. Nevertheless, we kept this possibility in mind and ticagrelor was switched to prasugrel.

Another rare aspect of our case was the occurrence of multiple CAA at the site of bifurcation stenting as demonstrated by the check angiography performed at six weeks of DES implantation. The predominant cause of CAA after DES implantation is postulated to be the occurrence of hypersensitivity to the DES polymer leading to a local vasculitis, many months after the DES implantation when the drug completely elutes from it [2,3]. In our case, Xience everolimus-eluting stents were used, which have a drug elution time of 120 days [18]. This argues against the possibility of polymer hypersensitivity being the predominant cause of CAA in our case, as our patient developed CAA within a few weeks. Another possible mechanism implicated in the development of CAA after DES implantation includes the mechanical injury caused to the vessel wall at the time of PCI due to the use of oversized balloons/stents, high-pressure balloon dilatations, or the use of atherectomy/laser angioplasty. Although it is not possible to entirely rule out the role of this mechanism in the causation of the CAA in our case, this possibility appears less likely, as the aneurysms occurring due to these mechanical factors tend to present early [2], and in our case the check angiography performed after 11 days of DES implantation had revealed features of stent thrombosis, with no hint of development of CAA.

A probable explanation for the development of CAA in our case appears to be related to the hyperinflammatory response due to COVID-19 illness, which could have led to the development of vasculitis, thus weakening the arterial wall. A vasculitis illness similar to Kawasaki’s disease, with resultant coronary aneurysm formation, has been reported in children with COVID-19-related inflammatory syndrome [19], and an inflammatory syndrome similar to that reported in pediatric population has also been reported in adults [20].

## Conclusions

The hypercoagulable and hyperinflammatory state associated with COVID-19 has important implications for cardiac patients, especially those undergoing complex coronary intervention. It may predispose these individuals to an increased risk of development of post-intervention complications including stent thrombosis and CAA formation. Further studies are needed in this field to assess the role of anticoagulant therapy combined with anti-platelet therapy in COVID-19 patients who have undergone a recent complex coronary intervention procedure. Also, the role of anti-inflammatory therapy in the prevention or treatment of CAA in these patients needs to be studied.
